# Gout

**Published:** 1900-04-07

**Authors:** 


### GOUT.

At the last meeting of the Medical Society the question of gout and its treatment came up again, two very interesting communications on the subject being read, one by Dr. Luff and the other by Dr. Bain, of Harrogate. The main object in Dr. Luff's communication was to show that while in all probability uric acid when first introduced into the blood existed solely as sodium quadriurate, which afterwards, in consequence of the instability of this salt, turned into the insoluble biurate, there was a period or stage in which this biurate existed in its gelatinous modification, and that if this were not eliminated in the urine it slowly or rapidly, according to circumstances, changed into the almost insoluble crystalline form which, by its deposition in the tissues, caused the gouty paroxysm. The chances of delaying or postponing the gouty paroxysm depended, then, very much upon our power of delaying the change of the biurate from its gelatinous to its insoluble form. Thus, we find the matter pushed on a step further. We used to think that it was the change from the quadriurate to the insoluble biurate that was the cause of the trouble; now we see that it is the change from the one modification of the biurate to the other. The results of Dr. Luff's researches, so far as treatment is concerned, were to show that for the purpose of delaying the conversion of the gelatinous biurate into the insoluble form the potassium salts were the most useful; that the lithium salts ranked next; and that piperazine and lysidine were not nearly so useful. He did not for a moment intend to argue that the beneficial effects of the salts of the alkali metals in the treatment of gout were to be solely gauged either by their solvent action on deposits of sodium biurate or by their inhibitory action on the conversion of the gelatinous into the crystalline form of this salt. Undoubtedly many of them were useful in the gouty state by their stimulating effect upon metabolism, by their remedial action upon the gastric and hepatic functions, by their diuretic action, and by diminishing the acidity of the urine, but he wished to point out that the oft-repeated statement that most of these drugs were useful for their great solvent action on gouty deposits was a loose and erroneous statement which it would be wiser to avoid in future.

